# Pain Management of Pediatric Musculoskeletal Injury in the Emergency Department: A Systematic Review

**DOI:** 10.1155/2016/4809394

**Published:** 2016-04-11

**Authors:** Sylvie Le May, Samina Ali, Christelle Khadra, Amy L. Drendel, Evelyne D. Trottier, Serge Gouin, Naveen Poonai

**Affiliations:** ^1^Faculty of Nursing, University of Montreal, Montreal, QC, Canada H3T 1A8; ^2^CHU Sainte-Justine Research Centre, Montreal, QC, Canada H3T 1C5; ^3^Women and Children's Health Research Institute, Edmonton, AB, Canada T6G 1C9; ^4^Department of Pediatrics, Faculty of Medicine & Dentistry, University of Alberta, Edmonton, Canada T6G 1C9; ^5^McGill University Health Centre, Montreal, QC, Canada H4A 3J1; ^6^Department of Pediatrics, Section of Emergency Medicine, Medical College of Wisconsin, Milwaukee, WI 53226, USA; ^7^Division of Emergency Medicine, Department of Pediatrics, Sainte-Justine Hospital (CHU Sainte-Justine), Montreal, QC, Canada H3T 1C5; ^8^Children's Hospital, London Health Sciences Centre, London, ON, Canada N6A 5W9; ^9^Schulich School of Medicine and Dentistry, London, ON, Canada N6A 5C1; ^10^Child Health Research Institute, London, ON, Canada N6C 2V5

## Abstract

*Background*. Pain management for children with musculoskeletal injuries is suboptimal and, in the absence of clear evidence-based guidelines, varies significantly.* Objective*. To systematically review the most effective pain management for children presenting to the emergency department with musculoskeletal injuries.* Methods*. Electronic databases were searched systematically for randomized controlled trials of pharmacological and nonpharmacological interventions for children aged 0–18 years, with musculoskeletal injury, in the emergency department. The primary outcome was the risk ratio for successful reduction in pain scores.* Results*. Of 34 studies reviewed, 8 met inclusion criteria and provided data on 1169 children from 3 to 18 years old. Analgesics used greatly varied, making comparisons difficult. Only two studies compared the same analgesics with similar routes of administration. Two serious adverse events occurred without fatalities. All studies showed similar pain reduction between groups except one study that favoured ibuprofen when compared to acetaminophen.* Conclusions*. Due to heterogeneity of medications and routes of administration in the articles reviewed, an optimal analgesic cannot be recommended for all pain categories. Larger trials are required for further evaluation of analgesics, especially trials combining a nonopioid with an opioid agent or with a nonpharmacological intervention.

## 1. Introduction

We have close to 20 years of evidence showing that analgesia for pediatric patients in the emergency department (ED) is suboptimal [1–6]. A recent medical record review of children presenting to an ED with acute musculoskeletal injury (MSK-I) confirmed that only 35% received an analgesic of any kind [7]. This is surprising, given the robust evidence that children experience both short- and long-term consequences when pain is undertreated [8–11].

Policy statements of pain and pediatric and emergency societies endorse the appropriate treatment of children's pain as a key part of ED clinical care [12–15]. Children with MSK-I (i.e., simple fracture or severe sprain) in the ED will likely experience maximal pain during the period between the occurrence of the fracture and the point of immobilization of the limb in a cast. During this period, the child will often undergo manipulation of the injured limb by a nurse, physician, medical trainee, and an X-ray technician [7, 12–16]. There is little doubt that sufficient analgesia would improve the child's experience [16]. Yet, there is no consensus as to what constitutes the best analgesic or combination of analgesics for this type of injury.

To our knowledge, there are no systematic reviews of interventions in pain relief for children presenting to the ED with a MSK-I. Our objectives were (a) to review the available randomized controlled trials related to the ED pain management of MSK-I in children and (b) to quantify the occurrence of any serious adverse events related to the use of the analgesics among the included studies. We aimed to assess the efficacy of the various interventions used and, thereby, identify which one(s) to recommend in the ED for addressing pediatric MSK-I related pain.

## 2. Methods

### 2.1. Study Design

We conducted a systematic review of randomized controlled trials of nonpharmacological (e.g., distraction and music), physical (e.g., splinting), and pharmacological (e.g., opioids, nonopioids, and nonsteroidal anti-inflammatory medications) interventions in children and adolescents under 19 years of age presenting to the ED with a musculoskeletal injury. Studies of both adult and pediatric participants were included if the authors were able to provide pediatric-specific data. We excluded studies that compared therapies for fracture reduction, pain management in patients with osteogenesis imperfecta, and pain management for postdischarge pain. Substudies of previously reported trials, crossover studies, and abstracts or conference proceedings of trials without sufficient information were excluded. The primary outcome was the risk ratio for successful reduction in pain scores. The secondary outcome was the occurrence of serious adverse events.

### 2.2. Search Strategy

We performed a systematic search for published and unpublished articles in accordance with the Preferred Reporting Items for Systematic Review and Meta-Analysis (PRISMA) guidelines [17] using the following databases: Cochrane Central Register of Controlled trials (CENTRAL) (1984–April 2015), MEDLINE (1984–April 2015) using the Ovid interface, PubMed (1975–2015), Embase (1988–April 2015), and ProQuest (1980–April 2015). The search strategy is described in the Appendix. In order to identify unpublished and ongoing trials, we searched numerous international trials registers and Google Scholar. Key journals and conference proceedings from major meetings were hand-searched from 2000 to 2015. Lastly, we contacted authors for further information and checked reference lists of all included trials. We restricted our search to articles written in either French or English.

### 2.3. Study Selection

Three review authors (SLM, SA, and SG) independently screened titles and abstracts using a standardized form with predefined eligibility criteria. Full-text articles were independently assessed by each of the three authors to determine whether or not they met inclusion criteria. Discrepancies were resolved by consensus. Selected studies were evaluated for methodological quality and appropriateness. Studies rejected at this stage were noted with reasons for exclusion. Authors of studies were contacted, if necessary, for clarification or missing data. Review authors were not blinded to author, institution, journal, or results of a study prior to its assessment.

### 2.4. Data Collection and Analysis

Data for each included study were extracted and recorded independently by three review authors (SLM, SA, and SG) on a standardized data extraction form. Any differences were resolved by discussion between review authors until consensus was reached. The data was entered into Review Manager (RevMan) software (version 5.1. Copenhagen: The Nordic Cochrane Centre, The Cochrane Collaboration, 2011). We collected data for study eligibility, randomization technique, interventions, age, type of intervention, dose, route, and cointerventions. For the primary and secondary outcomes, we collected pain scores, mean pain score difference, and occurrence of serious (life-threatening) adverse events. Any missing data were sought through communication with the study authors.

Risk ratios were calculated to evaluate treatment effects according to the studies retained for this review. For continuous outcomes, results were summarized using a mean difference and 95% confidence interval (CI). We measured the efficacy of the interventions, but not the number needed to treat to benefit (NNT) or number needed to treat to harm (NNH). The decision to pool the data was based on comparability of the analgesic agents and their dosing among studies.

### 2.5. Assessment of Methodological Quality

Studies that met inclusion criteria were graded independently for methodological rigour using the Cochrane Collaboration's Risk of Bias assessment tool [18] by three reviewers (SLM, AD, and NP). Studies were scored as* high*,* low*, or* unclear* risk of bias based on random sequence generation, allocation concealment, blinding of participants and personnel, blinding of outcome assessment, incomplete outcome data, selective reporting, and other biases. Discrepancies were resolved through discussion.

### 2.6. Dealing with Missing Data

**Figure 1:**
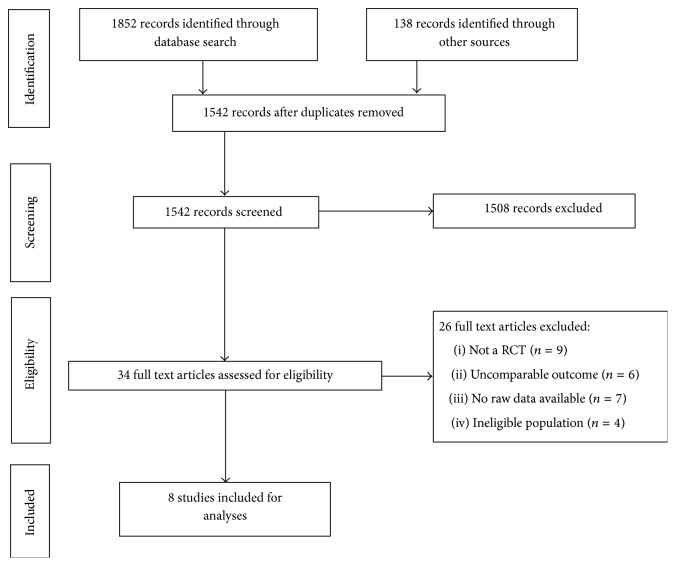
PRISMA Study Flow Diagram.

All authors were contacted for missing data. When data remained missing, the studies were not included and are indicated in the flow diagram (Figure 1) with “no raw data available” as the reason of exclusion.

## 3. Results

### 3.1. Study Selection

The PRISMA Study Flow Diagram (Figure 1) shows the flow of studies for this review. Of the 34 studies of potential relevance identified, 26 were excluded: nine were not randomized controlled trials [16, 19–26]; one was an adult-only study [27]; one included only two children over a total of 67 patients with no specific pediatric data available [28]; one was concerned with fracture reduction [29]; one was conducted with healthy volunteers [30]; six had data collected at the participant's home after discharge from the ED [31–36]. The remaining seven studies were excluded despite adequate methodology, because we were unable to obtain the raw data necessary to calculate their risk ratio, despite multiple efforts to obtain this information from the authors [37–43]. In total, eight studies were included [44–51].

### 3.2. Study Characteristics

Characteristics of included studies are presented in Table 1. The eight included studies provided data for 1169 children, aged 3–13 years [47, 48] and 5–18 years [44–46, 49–51]. Various analgesic agents were compared: codeine versus ibuprofen, ibuprofen versus acetaminophen, and acetaminophen versus codeine (three-arm study) [45]; acetaminophen/codeine versus ibuprofen [46]; diamorphine versus morphine [49]; fentanyl/ibuprofen versus ketamine/ibuprofen [48]; and ketorolac versus tramadol [51]. Two studies compared fentanyl to morphine [44, 47] and one study compared a combination of ibuprofen/codeine to ibuprofen/placebo [50].

Pain was assessed in the eight studies using various one-dimensional pain scales: Visual Analog Scale (VAS) [44, 45, 48, 50, 51], Color Analog Scale (CAS) [46], Wong-Baker faces pain scale (WBS) [47, 49], Faces Pain Scale-Revised (FPS-R) [48], and McGrath scale [51]. The primary outcome in all of the studies was the mean pain score difference.

### 3.3. Data Synthesis

Differences in analgesic agents studied precluded a meta-analysis. Only two studies [44, 47] used the same analgesic agents. A random-effects model was used for comparisons of compounded results.

### 3.4. Risk of Bias

**Figure 2:**
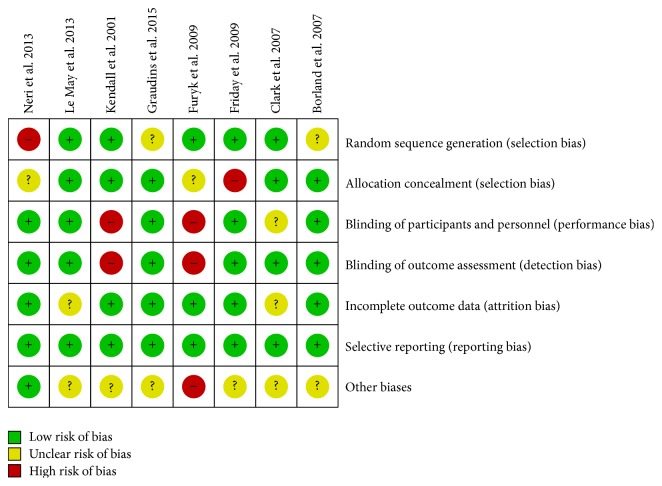
Risk of bias.

Figure 2 presents the risk of bias in the included studies according to the Cochrane Collaboration's Risk of Bias tool. The risk of bias was assessed by two independent raters (AD and NP) and disagreement was resolved through consultation with the first author (SLM). Overall, all included studies, except one [51], reported that their randomization sequence was adequately generated. This study had eligible patients randomly allocated by the physician, thus presenting a high risk of bias [51]. In five studies, allocation was adequately concealed [44–46, 48–50]. Blinding of participants and personnel was adequately performed in five studies [44, 46, 48, 50, 51]. Blinding of outcome assessors was adequately performed in six studies [44–46, 48, 50, 51]. Incomplete outcome data were adequately addressed in six studies [44, 46–49, 51]. Selective reporting bias was found low in all included studies. Only one study [47] presented a high risk of bias (other biases) because other analgesics besides the study analgesics were administered, during the study, at the discretion of the treating physician. Furthermore, the use of nonpharmacological strategies was not reported and may have led to baseline heterogeneity.

### 3.5. Pain Management

Each study reported results as the mean pain score difference. We also used the raw data provided by study authors to calculate the risk ratios (RR) between interventions for each study. Differences in the medication used among the eight studies precluded the use of meta-analysis techniques. Three studies [45, 48, 50] provided the data necessary for the calculation of their RR in their respective article. For the remaining five studies [44, 46, 47, 49, 51], data was provided upon request by each of the corresponding author or coauthors. In all of the included studies, there were no “true” control groups (group receiving no analgesia), as it would be considered ethically unacceptable to refrain from treating a child's pain.

We were able to combine two studies, as they both compared fentanyl (intranasal and nebulized) with morphine (intravenous) [44, 47]. No statistically significant heterogeneity (*I* ^2^ = 0%) was found and the RR was not significant (RR: 1.13; 95% CI: 0.93–1.38; *P* = 0.22) (Figure 2). The six other studies could not be pooled, as they studied different analgesics. The study by Friday et al. [46] compared the combination of acetaminophen-codeine to ibuprofen. The RR was not significant towards either group (RR: 1.00; 95% CI: 0.60–1.66; *P* = 0.99) (*n* = 66 children). Clark et al. [45] compared three different analgesics each one to the other, yielding three separate comparisons. In this study, ibuprofen was not considered as a control but having the same weight as acetaminophen and codeine. The RR of the first comparison between codeine and ibuprofen was not significant for either analgesic (RR: 1.30; 95% CI: 0.96–1.76; *P* = 0.09) (*n* = 200 children). For the second comparison, between ibuprofen and acetaminophen, the RR was in favour of ibuprofen and the difference was statistically significant (RR: 0.69; 95% CI: 0.50–0.96; *P* = 0.03) (*n* = 200 children). In the third comparison, between codeine and acetaminophen, the RR was not in favour of either analgesic (RR: 0.90; 95% CI: 0.63–1.28; *P* = 0.56) (*n* = 200 children). Le May et al. [50] compared a combination of ibuprofen-codeine to a combination of ibuprofen-placebo of codeine. The RR was not in favour of either combination (RR: 0.76; 95% CI: 0.45–1.29; *P* = 0.31) (*n* = 62 children). The study by Graudins et al. [48] compared intranasal fentanyl/oral ibuprofen to intranasal ketamine/oral ibuprofen. The RR was not in favour of either combination (RR: 0.96; 95% CI: 0.77–1.22; *P* = 0.76) (*n* = 68). The study by Kendall et al. [49] compared intranasal diamorphine with intramuscular morphine. The RR was not in favour of either analgesic (RR: 1.14; 95% CI: 0.81–1.59; *P* = 0.45) (*n* = 384). Finally, the study by Neri et al. [51] compared sublingual ketorolac to sublingual tramadol. No statistically significant difference was observed between analgesics (RR: 1.10; 95% CI: 0.99–1.22; *P* = 0.06) (*n* = 125).

The minimally clinically significant difference (MCSD) in analgesic effectiveness varied across studies depending on the scale used. One study [47] used a mean pain score difference > 1.0 for the WBS. On a 0 to 100 mm VAS, one study [44] used >13 mm and two studies [46, 48] used >20 mm, while one study [45] considered <30 mm as the pain score indicating adequate pain relief on that scale. Another study [50] considered 1.5 (standard deviation: 2.0) as a MCSD on a VAS that ranged from 0 to 10. Finally, on the same scale, another study [51] considered <5/10 as an adequate pain relief.

### 3.6. Serious Adverse Events

Only two serious adverse events were reported in the eight studies. Clark et al. [45] noted one important error in the preparation of the study medication (codeine), which led to the administration of fivefold higher dose to the patient. No adverse outcome was observed for this patient as the error was quickly identified and the patient received proper treatment (oral charcoal and monitoring in the ED). In the group receiving IM morphine, Kendall et al. [49] reported one episode of nausea and vomiting that they considered a serious adverse event of moderate intensity. The patient was admitted for a short period of observation but recovered spontaneously. No other serious adverse events occurred among the remaining 1167 participants, including the 783 (66.98%) patients who received opioid.

## 4. Discussion

A child's risk of sustaining a fracture before the age of 16 years ranges from 27 to 42%, [52–54] making MSK-I very common presentation to the ED [2, 55]. Unfortunately, the injury itself, as well as its assessment and treatment (i.e., radiographic tests and cast application), can cause significant pain and distress to a child. Children presenting to the ED with either a fracture or severe sprain often suffer from moderate-to-severe pain [33, 45, 56]. Current pain management, however, is recognized as suboptimal [7] and may be due in part to lack of clarity as to which medication to prescribe [57, 58]. The main objective of this systematic review was to identify the most effective interventions for reducing pain related to acute MSK-I. To our knowledge, this is the first systematic review of this subject. All of the studies retained were level I evidence: prospective randomized controlled trials with control groups (standard care or equivalent standard care). This review included eight randomized controlled trials [44–51] (1169 participants) assessing analgesics via different routes: oral, sublingual, intravenous, intramuscular, and intranasal.

### 4.1. Oral Pain Medications

One study demonstrated that ibuprofen was significantly more effective than acetaminophen [45] to relieve pain due to a simple fracture or severe sprain. Of note, only 52% of patients receiving ibuprofen achieved adequate pain relief, defined as <30 mm on a 100 mm Visual Analog Scale.

When codeine, alone, was compared to ibuprofen or acetaminophen [45], it did not provide better relief of pain. Moreover, even when codeine was combined with acetaminophen [46] or ibuprofen [50], these dual therapy combinations were considered equivalent to ibuprofen (10 mg/kg) alone [46]. The poor clinical performance for codeine can likely be explained by its genetic polymorphism-influenced metabolism, which can reduce its clinical effectiveness and influence its side effect profile [59–61]. Codeine, a prodrug, requires metabolizing to release morphine, its active analgesic component. The enzyme responsible for conversion of the prodrug to its active form is cytochrome P450-2D6. Approximately 50% of the North American Caucasian population has at least one reduced functioning allele for CYP2D6; this decreases the effectiveness of codeine for each affected individual [62–65]. Codeine has fallen out of favour as an analgesic for children because of this high interindividual variability and its association with drug-related deaths due to ultrarapid metabolizing [59, 66]. Currently, Health Canada recommends that no codeine-containing products should be used in any child less than 12 years of age [67]. In an effort to identify a reasonable agent to replace codeine, a recent study has compared oral morphine (0.5 mg/kg) to ibuprofen (10 mg/kg) for fracture pain in the post-ED setting [35]. Authors found no significant difference in analgesic efficacy between orally administered morphine and ibuprofen but noted that morphine was associated with a significantly greater number of side effects (56% versus 31%).

### 4.2. Intranasal Pain Medications

Three trials studied the use of intranasal fentanyl [44, 47, 48]. Two trials demonstrated that intranasal fentanyl was no different in efficacy compared to intravenous morphine to treat children with fractures [44, 47]. Larger sample sizes, with increased power, may show a significant difference between treatments, in the future. A third study compared intranasal fentanyl to intranasal ketamine (a dissociative anaesthetic) and demonstrated that the two were comparable in effectiveness for pain reduction, when combined with ibuprofen [48]. Similarly, another study [49] compared intranasal diamorphine (a semisynthetic moderate potency derivative of morphine) to intramuscular morphine and determined that they were associated with similar pain reduction in children with moderate-to-severe pain. Recent work has confirmed that intranasal diamorphine demonstrates no serious adverse events in over 200 children [68]. But another study [69] has shown an adverse event rate of 26.5%, which is in keeping with most other opioid medications. Intranasal pain medications have great intuitive appeal for clinicians, as they can be administered more quickly and without a potentially painful intravenous insertion or intramuscular injection. It is widely accepted that all intramuscular analgesic injections should be avoided in children when other routes are available [70]. Still, there remains limited evidence to definitively support intranasal pain medication use in children, at this time.

### 4.3. Sublingual Pain Medications

Sublingual medications have the appeal of rapid onset, can be used in children as young as six years of age, and preclude the need to swallow pills. A single study compared sublingual tramadol (a synthetic analogue of codeine and a weak u-opioid receptor agonist) to sublingual ketorolac (a nonsteroidal anti-inflammatory drug) [51]. While this study failed to demonstrate that either drug was more effective than the other, the results trended strongly towards ketorolac being more clinically effective. A larger trial would likely be able to clarify this. As with ibuprofen and codeine, a nonsteroidal anti-inflammatory medication appears to be outperforming weak opioid, even via the sublingual route.

### 4.4. Nonpharmacological Techniques

Few studies have addressed the efficacy of nonpharmacological interventions (e.g., splinting, cold, and distraction techniques) to relieve children's pain related to MSK-I. We did not find a single trial specifically studying the analgesic effect of splinting injured limbs. We identified one nonpharmacological intervention study in our search [25] but it could not be included in the present review as it was not a randomized controlled trial. In this study, Tanabe et al. [25] demonstrated that all groups (standard care, standard care plus 10 mg/kg ibuprofen, and standard care plus distraction) achieved a statistically significant reduction in pain at 30 minutes; the largest decrease in pain ratings was for those who received standard care plus distraction in the form of music and/or toys. A recently updated Cochrane Database systematic review of psychological interventions for procedural pain has confirmed that there is strong evidence for the use of distraction, in this setting [71]. Given that there is minimal risk involved in the use of distraction, it stands to reason that its benefits as an adjuvant to pharmacological therapy are both feasible and likely beneficial. Recognizing that implementing trials that study the efficacy of nonpharmacological interventions are extremely challenging and that nonpharmacological interventions, alone, may not be adequate, their utility could be studied in the context of an adjuvant to pharmacological therapy.

### 4.5. Perspectives on Future Studies

Current research would suggest that ibuprofen would be the recommended first choice for mild-to-moderate acute musculoskeletal pain. However, it may not be adequate, when used alone, for moderate-to-severe pain. The intranasal route for pain medication administration has recently become quite popular, with four of our eight included studies exploring this modality. While intranasal fentanyl appears to be promising, a large scale randomized controlled trial would be required to confirm its efficacy, as it is rapidly gaining popularity within the clinical realm, despite limited evidence. Further, because of its short-acting effect, it would probably require more than one dose to provide proper relief for a MSK-I. Moreover, once clinical efficacy and adverse effect profiles are better understood, further studies should also focus on the cost-effectiveness of these medications. While the cost of an individual dose of an oral medication (ibuprofen $0.38 for 400 mg suspension dose and acetaminophen $0.42 for 500 mg suspension dose) versus intranasal fentanyl ($0.45 for a 100 mcg ampoule) is comparable, the single patient-use atomizer required for intranasal delivery can be costly ($3.50–$5.00 per atomizer).

Finally, we would also recommend that future studies on pain management of MSK-I with children adopt a standardized primary outcome to assess pain management efficacy. Studies retained for this review had various primary outcomes of MPS from 13 to less than 30 mm on a 0–100 mm VAS as well as less than 5 over 10 on a 0–10 VAS and a difference of 2 cm on a 0–10 cm CAS. This wide range of primary outcomes makes it hard to properly compare results between studies. Further, when comparing analgesics in a trial, one should make sure that their respective delay for onset and peak of action are similar as well as setting a study time measure for the primary outcome accordingly. For instance, in Friday's trial [46], measure of the primary outcome was set at 40 minutes after analgesic administration. Yet, all of the study medications for this trial were oral analgesics (ibuprofen, acetaminophen, and codeine) requiring at least 60 minutes to reach their peak of action. It was then kind of expected that the authors mentioned that they did not find any significant difference between groups at 40 minutes. They concluded by stating that ibuprofen was comparable in efficacy to the combination of acetaminophen and codeine, which is misleading. In fact, their results showed a positive trend in pain reduction in the group who received acetaminophen and codeine at 60 minutes.

### 4.6. Limitations

There were some limitations inherent to this systematic review. Firstly, we were unable to pool results and generate a summary statistic due to the disparity in the analgesics chosen and variations in the way intergroup differences in efficacy were reported. Secondly, the absence of response from some authors and the nonavailability of the data from several corresponding authors of studies meeting inclusion criteria prevented us from presenting all available studies regarding pain management of children presenting to the ED with MSK-I. Finally, the search was limited to articles published in either English or French, and no grey literature was searched for non-English language articles. The search was limited to randomized controlled trials and therefore excluded quasi-experimental studies and other designs.

## 5. Conclusion

In conclusion, based on our review of the currently available evidence, no one specific analgesic agent or intervention has been clearly identified as the optimal choice in the ED for all scenarios of pain management related to pediatric MSK-I. The ideal analgesic agent(s) for moderate-to-severe pain and the utility of nonpharmacological interventions in the pediatric population have yet to be determined. Our review results underscore the need for larger trials with a standardized primary outcome, to generate strong evidence for pain treatment of children presenting to the ED with a MSK-I. Finally, there is an urgent need to definitively address the issue of safety of commonly used analgesic agents in children, with special emphasis on opioid medications.

## Figures and Tables

**Table 1:** Characteristics of included studies.

Author	Participants	Intervention^*∗*^	Main outcome	Findings
Borland et al. 2007 [44]	*N* = 67 children with acute long-bone fractures Age: 7 to 15 years	Group 1: *n* = 33, IN fentanyl 1.4 *μ*g/kg Group 2: *n* = 34, IV morphine 0.1 mg/kg	Difference in MPS greater than 13 mm on the VAS at 5 minutes after analgesic administration	No significant differences in pain scores between groups at all study points

Clark et al. 2007 [45]	*N* = 300 children with acute musculoskeletal pain Age: 6 to 17 years	Group 1: *n* = 100, acetaminophen 15 mg/kg Group 2: *n* = 100, ibuprofen 10 mg/kg Group 3: *n* = 100, codeine 1 mg/kg	Difference in MPS greater than 15 mm on the VAS at 60 minutes after analgesic administration	(i) Ibuprofen group showed significantly greater reduction in VAS but only 52% of participants in this group received adequate analgesia (VAS < 30 mm)

Friday et al. 2009 [46]	*N* = 66 children with fractures or dislocations Age: 5 to 17 years	Group 1: *n* = 34, ibuprofen 10 mg/kg, (max 400 mg) Group 2: *n* = 32, acetaminophen + codeine 15 mg/kg and 1 mg/kg (max 60 mg codeine)	Difference in MPS greater than 2 cm on the CAS at 40 minutes after analgesic administration	Equivalent analgesic effectiveness of both agents at 40 minutes

Furyk et al. 2009 [47]	*N* = 73 children with suspected limb fractures Age: 4 to 13 years	Group 1: *n* = 36, nebulized fentanyl 4 *μ*g/kg Group 2: *n* = 37, IV morphine 0.1 mg/kg	Difference in mean pain score greater than 1 face on the WBS at 15 and 30 minutes after analgesic administration	(i) Significantly decreased pain scores in both groups at all study time points (ii) No significant differences in pain scores between groups

Graudins et al. 2015 [48]	*N* = 73 children with limb injury; weighing < 50 kg Age: 3 to 13 years	Group 1: *n* = 36, IN ketamine 1 mg/kg + ibuprofen 10 mg/kg Group 2: *n* = 37, IN fentanyl 1.5 *μ*g/kg + ibuprofen 10 mg/kg	Median reduction in pain 30 min after analgesic administration FPS-R for children aged 3 to 6 and VAS for children 7 years and older	Similar pain reduction in both groups

Kendall et al. 2001 [49]	*N* = 384 children with a clinical fracture of an upper or lower limb in the ED Age: 3 to 16 years	Group 1: *n* = 191, IN diamorphine spray 0.1 mg/kg Group 2: *n* = 193, IM morphine 0.2 mg/kg	Pain score on WBS at 30 minutes after analgesic administration	(i) Both medications significantly reduced pain (ii) Onset of pain relief was faster in the IN spray group than IM (lower pain scores at 5, 10, and 20 minutes) (iii) No difference between groups at 30 minutes

Le May et al. 2013 [50]	*N* = 81 children with limb trauma Age: 6 to 18 years	Group 1: *n* = 41, ibuprofen 10 mg/kg + codeine 1 mg/kg (max 60 mg) Group 2: *n* = 40, ibuprofen 10 mg/kg (max 600 mg) + placebo	Difference in MPS of 20 mm on the VAS at 90 minutes after analgesic administration	No significant differences in mean pain scores between groups at all study time points

Neri et al. 2013 [51]	*N* = 125 children with suspected fracture or dislocation Age: 4 to 17 years	Group 1: *n* = 60, ketorolac (SL) 0.5 mg/kg Group 2: *n* = 65, tramadol (SL) 2 mg/kg	McGrath scale for children up to 6 years and VAS for those older than 6 years. Primary outcome was MPS < 5/10 on a 0–10 VAS at 120 minutes.	(i) Significant reduction in mean pain scores in both groups (ii) No significant differences in pain scores between groups at 120 minutes (iii) Rescue dose of paracetamol-codeine administered in 2/60 children in the ketorolac group versus 8/65 in the tramadol group (not significant)

^*∗*^Note: medications were given orally, except where otherwise indicated.

CAS: Color Analog Scale; IM: intramuscular; IN: intranasal; IV: intravenous; MPS: Mean Pain Score; RCT: Randomized Controlled Trial; SL: sublingual; VAS: Visual Analog Scale; WBS: Wong-Baker faces pain scale.

## Appendix

*Ovid Search Strategy*. Database: EBM Reviews-Cochrane Central Register of Controlled Trials, Embase, Ovid MEDLINE(R)

1. PAIN/
2. pain$ or discomfort.ti,ab.
3. Exp ANALGESICS
4. analges$.ti,ab.
5. OR/(1)–(4)
6. FRACTURES, BONE/
7. FRACTURES, CLOSED/
8. Exp RADIUS FRACTURES
9. HUMERAL FRACTURES/
10. SHOULDER FRACTURES/
11. ((simple or buckle or greenstick) adj6 fracture$).ti,ab.
12. ((radius or ulna or forearm or limb$ or arm$ or leg$ or musculoskeletal or musculo-skeletal or orthopaedic$ or orthopaedic$) AND (fracture$ or trauma$ or injur$)).ti,ab.
13. OR/(6)–(12)
14. Accident and Emergenc$ or emergency department$ or emergency room$ or A&E.ti,ab.
15. (5) AND (13) AND (14).

*Pubmed Search*. Search ((((((A&E[Title/Abstract]) OR emergency room$[Title/Abstract]) OR emergency department$[Title/Abstract]) OR ((Accident and Emergenc$[Title/Abstract])))) AND (((((((((((radius or ulna or forearm or limb$ or arm$ or leg$ or musculoskeletal or musculo-skeletal or orthopaedic$ or orthopaedic$) AND (fracture$ or trauma$ or injur$))[Title/Abstract]))) OR ((((simple or buckle or greenstick) adj6 fracture$)[Title/Abstract]))) OR SHOULDER FRACTURES/) OR HUMERAL FRACTURES/) OR Exp RADIUS FRACTURES) OR FRACTURES, CLOSED/) OR FRACTURES, BONE/)) AND ((((analges$[Title/Abstract]) OR Exp ANALGESICS) OR ((pain$[Title/Abstract]) OR discomfort[Title/Abstract])) OR PAIN/).

## Additional Points

Musculoskeletal injury (MSK-I) in children is very common and almost universally painful. Pain management of MSK-I in the emergency department (ED) varies greatly between hospitals. This paper is a systematic review of the current pain management for children presenting to the ED with MSK-I. Eight randomized controlled trials met inclusion criteria and provided data on 1169 children. Due to heterogeneity of medications among the studies retained, an optimal analgesic cannot be presently recommended. Current research would suggest ibuprofen as first choice for mild-to-moderate musculoskeletal pain. However, there is no evidence for its superiority when used alone for moderate-to-severe pain.
